# Clinical efficacy of losartan potassium combined with alprostadil in early diabetic nephropathy and its effect on renal function

**DOI:** 10.12669/pjms.41.6.10796

**Published:** 2025-06

**Authors:** Xiaoli Gong, Jihong Liu, Huidan Guo, Wei Zhang

**Affiliations:** 1 Xiaoli Gong Department of Nephrology, Heji Hospital Affiliated to Changzhi Medical College, Changzhi 046000, Shanxi, P.R. China; 2 Jihong Liu Department of Nephrology, Heji Hospital Affiliated to Changzhi Medical College, Changzhi 046000, Shanxi, P.R. China; 3 Huidan Guo Department of Nephrology, Heji Hospital Affiliated to Changzhi Medical College, Changzhi 046000, Shanxi, P.R. China; 4 Wei Zhang Department of Nephrology, Heji Hospital Affiliated to Changzhi Medical College, Changzhi 046000, Shanxi, P.R. China

**Keywords:** Alprostadil, Diabetic Nephropathy, Losartan Potassium, Renal Function

## Abstract

**Objective:**

To examine the effectiveness of the combination of losartan potassium and alprostadil in managing early diabetic nephropathy(DN) among patients and to determine its impact on renal function.

**Method:**

This was a retrospective comparative study. Eighty six patients with early DN treated in Heji Hospital Affiliated to Changzhi Medical College during March 2020 to March 2023 were selected and randomly assigned into two groups: the routine group(n=43) which received alprostadil therapy, and the intervention group(n=43) which received a combination of alprostadil and losartan potassium therapy, using an electronic grouping table. Compared the clinical efficacy, inflammatory indexes, renal function, and incidence of adverse drug reactions prior to and after treatment between the two groups.

**Results:**

The intervention group demonstrated a significantly higher clinical efficacy when compared to the routine group [97.67% vs. 86.05%, *P*< 0.05]. Prior to treatment, there were no considerable contrasts in levels of SCr, BUN, UP, CRP, and TNF-a between the two groups(*P*> 0.05). After treatment for 14 days, SCr, BUN, UP, CRP and TNF-a levels all reduced in both groups compared with those before treatment, which was more significant in the intervention group(*P*< 0.05). There was no meaningful contrast in the occurrence of relevant negative drug responses between the two groups during the 14 days treatment period (P > 0.05).

**Conclusion:**

Losartan potassium combined with alprostadil in the treatment of early DN may improve renal function and inflammatory indexes in patients, with significant clinical efficacy but no increased adverse drug reactions. Moreover, it presents high safety of treatment.

## INTRODUCTION

Type-2 diabetes mellitus (T2DM) is a prevalent endocrine disease in clinical practice. The incidence of T2DM has been on the rise in China due to rapid economic development and an improvement in the quality of life. It has become a significant public health concern that affects the overall health and quality of life of individuals.^1^,^2^ The harm of T2DM lies in continuous high blood glucose of the body, which damages various systems of the body, including important organs such as the heart, brain and kidney. Among them, diabetic nephropathy (DN) is one of the common complications.^3^,^4^ DN patients have no typical clinical signs at the early stage. Without timely diagnosis and treatment, continuous proteinuria can damage the renal tubules and interstitium as the occurrence and development of the disease, eventually causing renal failure, which will seriously affect the life, health, quality of life and economic burden of patients.

It has been pointed out that DN is reversible at the early stage, and early intervention can reverse the damaged renal function and prevent the sustained damage to the kidney.^5^ Alprostadil can improve renal microcirculation and renal function to a certain extent. Losartan potassium is an angiotensin II receptor antagonist (ARB), which can dilate the body’s capillaries, reduce intraglomerular pressure and also protect the kidney.^6^,^7^ The present study aimed to evaluate the effectiveness of the combination therapy of losartan potassium and alprostadil in managing early DN among patients, and to determine its impact on renal function.

## METHODS

This was a retrospective comparative study. A total of 86 patients diagnosed with early DN and treated at Heji Hospital Affiliated with Changzhi Medical College from March 2020 to March 2023 were included in the study were randomly splited into routine group(alprostadil therapy, n = 43) and intervention group(alprostadil combined with losartan potassium therapy, n= 43) using an electronic grouping table.

### Ethical approval:

The study received approval from the Institutional Ethics Committee at Heji Hospital Affiliated with Changzhi Medical College (No.:2020010; date: 20 January, 2020). Furthermore, all participants provided their written informed consent.

### Inclusion criteria:

- Pcombined with urinary microprotein detection.^8^
- Patients with indications for losartan potassium and alprostadil therapy.
- Patients with signed informed consent after explaining the treatment plan and research measures to the patients and their families.

### Exclusion criteria:

- Chronic renal failure, severe urinary tract infection, acute renal injury and renal tumor.
- Severe systemic infection and immune dysfunction.
- Contraindications and allergy to this medication.

The routine group was given routine treatment. According to exercise tolerance, the patients were guided to take a walk, practice Tai Chi, and eat low sugar and low protein diets, so as to avoid raising the blood glucose level and increasing the burden on the kidney. Based on blood glucose level and insulin resistance (IR), appropriate hypoglycemic drugs, including oral hypoglycemics and insulin, were selected to control stable blood glucose, with the target blood glucose degree of fasting blood glucose < 7.0 mmol/L. For the patients complicated with hypertension, stable blood glucose level was actively controlled, and for those with hyperlipidemia, stable blood lipid level was controlled. All patients underwent sputum culture + drug sensitivity test before treatment. Alprostadil injection (10 ug, Xi’an Libang Pharmaceutical Co., Ltd., Guoyao Zhunzi: H20103101) was added into 100 ml 0.9% sodium chloride injection for intravenous drip, once a day, for 14 consecutive days. On this basis, the intervention group was orally administrated with losartan potassium (Hangzhou MSD Pharmaceutical Co., Ltd., Guoyao Zhunzi: J20180054), 100 mg/time, 1 time/days, for 14 consecutive days. The adverse reactions such as lactic acidosis, gastrointestinal reaction, hypoglycemia and renal insufficiency should be observed during medication, and the hypoglycemic program and symptomatic treatment should be timely adjusted.

### Observation indexes:

*Efficacy evaluation:* Efficacy was evaluated according to the changes in relevant clinical indexes 14 days after treatment. Remarkably effective: the degrees of blood glucose, creatinine and urinary microalbumin were improved by > 75%; effective: the levels of blood glucose, urinary microalbumin and creatinine were improved by 25%~75%; ineffective: the degrees of blood glucose, creatinine and urinary microalbumin were improved by < 25%.^9^

*Renal function:* Fasting venous blood samples of 5mL were collected from the patients’ elbow prior to treatment and 14 days after treatment. The blood samples were collected in anticoagulant tubes and sent to the laboratory department for analysis of the degree of blood urea nitrogen (BUN) and serum creatinine (SCr) and using enzyme-linked immunosorbent assay (ELISA).Additionally, a 24 hours urine sample was collected from each patient to measure the degree of 24 hours urinary protein (UP).

*Inflammatory indexes:* Before and 14 days after treatment, 5mL fasting venous blood from the patients’ elbow was collected and sent to the laboratory department. The levels of C-reactive protein (CRP) and tumor necrosis factor-a (TNF-a) were detected and compared between the two groups by chemiluminescent immunoassay.

*Adverse drug reactions:* Before and during 14 days treatment, the incidence of adverse reactions such as gastrointestinal discomfort, chest pain, fatigue, dizziness and headache were compared between the two groups.

*Statistical Analysis:* The statistical analysis was conducted utilizing SPSS 24.0 application. Age, diabetes’ course, course of DN, renal function-related indexes and inflammatory indexes following the normal distribution were conveyed as (*(x̄ ± S)*) and analyzed using the *t* test. Sex, efficacy and the incidence of unfavorable drug responses were expressed as a number(n,%) and tested via χ^2^ test. α = 0.05 was thought statistically to be substantial.

## RESULTS

The clinical data showed no statistical significance between the two groups (*P* > 0.05), as seen in Table-I. In Table-II demonstrated that the clinical efficacy in the intervention group was significantly superior to the routine group [97.67% vs. 86.05%, *P*<0.05]. There was no statistically meaningful contrast in the levels of SCr, BUN, and UP between the two groups before treatment (P>0.05), as shown in Table-III. After 14 days of treatment, both groups showed a decrease in SCr, BUN, and UP levels compared to their respective levels prior to treatment; however, the reduction was more prominent in the intervention group (P<0.05). Prior to therapy, no statistical substance was detected in TNF-a degree or CRP between the two groups (P> 0.05). After therapy for CRP, TNF-a degrees and 14 days declined in both groups in comparison with those prior to therapy, which was more apparent in the intervention group (P<0.05), as seen in Table-IV. Table-V illustrates that there was no statistically meaningful contrast in the incidence of relevant adverse drug reactions between the two groups during the 14 days treatment period (P>0.05).

**Table-I:** Comparison of the clinical information.

Group	n	Male/Female (n)	Age (year)	Course of diabetes (year)	Course of DN (year)
Routine group	43	25/18	46.56 ± 6.58	6.47 ± 1.70	1.26 ± 0.44
Intervention group	43	22/21	46.14 ± 5.92	6.33 ± 1.73	1.16 ± 0.37
*χ*^2^/t	-	0.422	0.310	0.378	1.055
*P*	-	0.516	0.757	0.706	0.295

**Table-II:** Comparison of the clinical effectiveness [n (%)].

Group	n	Remarkably effective	Effective	Ineffective	Overall effective rate (%)
Routine group	43	22	15	6	86.05
Intervention group	43	30	12	1	97.67
*χ* ^2^	-				3.888
*P*	-				0.049

**Table-III:** Comparison of renal function (*x̄* ± *s*).

Group	n	SCr (umol/L)		BUN (mmol/L)		UP (g/24 hours)	
		Prior to treatment	Treatment for 14 days	Before treatment	Treatment for 14 days	Before treatment	Treatment for 14 days
Routine group	43	215.25 ± 39.30	168.45 ± 28.33*	12.95 ± 2.21	9.55 ± 2.00*	2.84 ± 0.68	1.52 ± 0.49*
Intervention group	43	213.87 ± 41.08	122.32 ± 26.97*	12.63 ± 2.89	8.13 ± 1.91*	2.91 ± 0.79	1.14 ± 0.37*
*t*	-	0.159	7.733	0.575	3.364	-0.478	3.990
*P*	-	0.874	0.000	0.567	0.001	0.634	0.000

***Notes:***

* Compared with that of prior to treatment, *P* < 0.05.

**Table-IV:** Comparison of inflammatory indexes (*x̄* ± *s*).

Group	n	CRP (mg/L)		TNF-a (ng/L)	
		Before treatment	Treatment for 14 days	Before treatment	Treatment for 14 days
Routine group	43	55.85 ± 10.83	17.37 ± 3.48*	46.55 ± 11.46	29.73 ± 8.63*
Intervention group	43	54.97 ± 11.42	13.16 ± 2.96*	46.95 ± 12.26	21.55 ± 6.40*
*t*	-	0.369	6.054	-0.156	5.000
*P*	-	0.713	0.000	0.876	0.000

***Notes:***

* Compared with before treatment, *P* < 0.05.

**Table-V:** Comparison of the occurrence rate of adverse drug reactions [n (%)].

Group	n	Gastrointestinal discomfort	Chest pain	Fatigue	Dizziness and headache
Routine group	43	1 (2.33)	0 (0.00)	2 (4.65)	1 (2.33)
Intervention group	43	0 (0.00)	1 (2.33)	1 (2.33)	1 (2.33)
*χ* ^2^	-	1.012	1.012	0.345	0.000
*P*	-	0.314	0.314	0.557	1.000

## DISCUSSION

Our results showed that losartan potassium integrated with alprostadil in treating patients with early DN significantly reduced the level of inflammatory indexes (*P* < 0.05). Some scholars also treated patients with early DN by alprostadil combined with losartan potassium, which significantly reduced non-infectious inflammatory markers and improved their renal function.^10^,^11^ In this study, no significant difference was found in relevant adverse drug reactions between the two groups during the treatment (*P* > 0.05), proving the combination of the two drugs has high safety. Adverse drug reactions are important factors affecting the therapeutic efficacy and safety. Losartan potassium and alprostadil are mainly used to strengthen the protection of patients’ renal function during treatment, the low incidence of relevant adverse drug reactions indicates its high safety in treating DN patients. T2DM is an endocrine metabolic disorder, which is related to many factors such as diet, lifestyle, obesity and heredity. In recent years, its incidence has increased year by year, and tends to be younger in clinic.^12^,^13^ T2DM patients are characterized by the main clinical sign of elevated blood glucose. Continuous high blood glucose level in the body affects not only endocrine and metabolism, but also blood vessels and nerves of the patients.^14^ A study has pointed out that the incidence of complications in T2DM patients is high, which can damage various systems and multiple vital organs of the body.^15^ Common complications secondary to T2DM include peripheral neuropathy, large and small vascular injuries, renal impairment and retinal damage, which are important causes affecting the health and quality of life of the patients and endangering their lives.^16^ DN is one of the common complications in diabetic patients, mainly manifested as elevated proteinuria and creatinine in clinic.

It has been shown that in the early stage of DN, due to the strong compensatory ability of the kidney, renal injury can be reversed and the sustained damage to the kidney can be ended, which is the important timing for clinical intervention.^17^ Therefore, some researchers have suggested that early DN is a period for detecting renal impairment in diabetic patients, and also an important timing for clinical intervention to reverse renal impairment.^18^ Alprostadil is a commonly used renal function-protecting drug in clinic, and its main component is prostaglandin (PG), which is a vasoactive substance. After acting on the body, it mainly dilates microarteries, which can increase the content of cyclic adenosine monophosphate in the smooth muscle of renal microarteries and promote vasodilation, thus improving renal microcirculation and increasing renal blood flow.^19^ Some researchers treated patients with early renal impairment using alprostadil, which significantly improved their renal function and reduced the level of microalbuminuria.^20^

Additionally, a pharmacological study has pointed out that the main active component of PG in alprostadil is PG E1, which is a lipid microsphere carrier with strong targeting effect, and can deliver drugs to the kidney, improve drug concentration in the kidney, and then enhance the pharmacological effect.^21^ Clinical research has revealed that the renin-angiotensin-aldosterone system plays a crucial role in renal injury, and the use of angiotensin II receptor antagonists has been found to be effective in preventing the occurrence and progression of DN.^22^ As a result, when creatinine allows, DN patients are mostly treated with adjuvant therapy using angiotensin II receptor antagonist in clinic to reduce the degree of renal impairment and improve the prognosis of patients.^23^ In the present study, renal function-related indexes were improved more significantly in patients with early DN treated with losartan potassium combined with alprostadil compared with those treated by alprostadil alone, suggesting more significant clinical efficacy (*P* < 0.05). Losartan potassium is a novel angiotensin II receptor antagonist in clinic.

A study has confirmed that losartan potassium therapy for DN patients can effectively reduce renal microvascular resistance, decrease renal capillary pressure and increase residual nephron perfusion, thereby improving the renal function of patients, and delaying the occurrence and development of the disease.^24^ In addition, research has indicated that the concurrent administration of losartan potassium and alprostadil for the treatment of early DN patients can substantially enhance renal function by reducing renal vascular resistance and improving renal microcirculation.^25^ The occurrence and progression of DN are heavily influenced by inflammatory factors and mediators. It is believed that high blood glucose level in diabetic patients can cause metabolic disorders and hemodynamic disorders. Furthermore, endocrine and metabolic disorders in the body can stimulate the elevation of inflammatory factors, which can act on important organs of the body, thus increasing IR and aggravating renal impairment.^26^

### Limitations

It includes small sample size is small and that the follow-up period is short. In the future, our research group will continue to carry out further study based on the expanded sample size and prolonged follow-up period, so as to benefit more patients via evaluating the advantages and disadvantages of the proposed intervention program more objectively.

## CONCLUSIONS

Losartan potassium combined with alprostadil in the treatment of early DN can improve renal function and inflammatory indexes in patients, with significant clinical efficacy but no increased adverse drug reactions. Moreover, it presents high safety of treatment.

### Authors’ Contributions:

**XG** and **JL:** Designed this study and prepared this manuscript, are responsible and accountable for the accuracy or integrity of the work

**HG:** Collected and analyzed clinical data. Critical Review.

**WZ:** Acquisition, analysis, or interpretation of data and draft the manuscript.

All authors have read and approved the final version.
